# Comparison of cardiopulmonary function in patients with scoliosis at rest and during exercise: a case-control study

**DOI:** 10.3389/fsurg.2026.1931071

**Published:** 2026-10-13

**Authors:** Bo Zhou, Zhibo Song, Ni Bi, Li Zhang, Zhiyue Shi, Tao Li, Chengquan Cao, Zhi Zhao, Yingsong Wang

**Affiliations:** Department of Orthopedics, The Second Affiliated Hospital of Kunming Medical University, Yunnan International Joint Research & Development Center for Innovative Spinal Deformity Prevention and Treatment Technologies, Kunming, China

**Keywords:** cardiopulmonary exercise testing, congenital scoliosis, preoperative risk stratification, pulmonary function, scoliosis, thoracic height

## Abstract

**Background:**

Preoperative cardiopulmonary assessment in scoliosis is typically dominated by resting spirometry and the Cobb angle, yet resting tests may understate functional impairment and curve magnitude predicts it only weakly. This study integrated resting echocardiography, static pulmonary function testing, CT lung volume, and cardiopulmonary exercise testing (CPET) in the same preoperative patients to characterize cardiopulmonary impairment and to separate the structural determinants of lung volume from those of exercise reserve.

**Methods:**

In this single-center case-control study, 33 consecutive preoperative patients with scoliosis [21 adolescent idiopathic scoliosis (AIS), 12 congenital scoliosis (CS); aged 10–20 years] were compared with 20 age- and sex-matched healthy controls. Predicted spirometric values were derived from arm span with the GLI Global equations. Analyses used effect sizes with false-discovery-rate (FDR) correction, body-size-adjusted hierarchical regression, and cross-validated ROC analysis.

**Results:**

Resting cardiac function was preserved (left ventricular ejection fraction, cardiac output and cardiac index all non-significant after FDR), whereas restrictive ventilation and exercise reserve were reduced: FVC 72.0% vs. 92.8% predicted (q < 0.001), peak oxygen uptake 21.8 vs. 29.6 mL/min/kg (q < 0.001), O₂ pulse 5.9 vs. 9.7 mL/beat (q < 0.001), and VE/VCO₂ slope 32.4 vs. 27.5 (q < 0.001). Among 13 patients normal at rest, 10 (77%) were abnormal on CPET. Thoracic height independently predicted restrictive impairment beyond the Cobb angle, kyphosis, and body size (FVC %predicted B = 3.65 per cm, 95% CI   1.64–5.65; AUC 0.90, cross-validated 0.84; threshold 19.6 cm), whereas no deformity parameter independently predicted exercise capacity. At matched thoracic height, congenital scoliosis had lower CT lung volume than idiopathic scoliosis (1,881 vs. 2,356 cm^3^; *p* = 0.030).

**Conclusion:**

Preoperative scoliosis is characterized by preserved resting indices but a frequently occult reduction in exercise reserve, including an exercise cardiac limitation revealed by O₂ pulse that resting echocardiography does not show. Thoracic height is a body-size-independent predictor of restrictive lung volume with a clinically usable threshold, while exercise reserve is multifactorial and requires direct CPET assessment. The two dimensions of preoperative cardiopulmonary risk should therefore be measured separately.

## Background

Scoliosis is a three-dimensional deformity of the spine and rib cage, and that second fact is what makes it a cardiopulmonary problem as much as an orthopedic one. As the thorax twists and narrows, chest-wall compliance falls and the lungs lose room to expand, producing the restrictive ventilatory pattern long recognized as a determinant of perioperative risk in scoliosis surgery (1, 2); respiratory events remain the commonest major non-neurological complication of deformity correction, with non-idiopathic etiology and a low forced vital capacity among their risk factors (3, 4). The burden grows with the curve, and it does so without a convenient cutoff: a meta-regression of 126 studies and 8,723 patients found pulmonary function declining gradually across the whole range of Cobb angles, from below 20° to above 120°, with no threshold below which the lungs can be considered reliably safe (5). Progressive thoracic deformity stiffens the chest wall, alters trunk symmetry and impairs physical function and quality of life, and in its most severe and long-standing forms carries measurable excess respiratory mortality (6, 7). The heart is not a bystander either. A meta-analysis of 9,554 patients reported cardiac abnormalities in roughly one in thirteen, including pulmonary hypertension in 16.8%, and concluded that careful cardiovascular evaluation should precede surgery (8). The physiological cost of scoliosis is therefore integrated across heart and lung, and measuring it accurately before an operation matters for risk stratification.

How that cost is measured, however, has split into two literatures that rarely meet. One has worked to predict pulmonary function from the shape of the deformity, moving from the plain Cobb angle to three-dimensional descriptors of rotation and rib-cage deformity (9, 10), and on to CT reconstruction and machine-learning models (11, 12); the most recent can explain about half the variance in forced vital capacity from radiographic rotation indices alone (9), while a careful head-to-head still finds a simple 2D Cobb angle the better predictor of FEV₁ (13). Conversely, after correction of severe deformity by vertebral column resection, the improvement in pulmonary function bears no relation to the degree of Cobb correction (14), which underlines that curve magnitude is an imperfect proxy for the thoracic constraint. What these studies share is their endpoint. They predict spirometry measured at rest, and they seldom look past the lungs. The other literature starts from exercise, and it keeps arriving at the same uncomfortable conclusion, that resting spirometry understates the true functional deficit. Aerobic capacity on cycle-ergometer cardiopulmonary exercise testing is consistently reduced and largely independent of the Cobb angle (15–18), and a recent systematic review put the shortfall at roughly twenty to twenty-five percent below reference while naming VO₂max the most integrative marker of physiological burden and cautioning that spirometry can stay deceptively normal (19); treadmill studies of children treated for early-onset scoliosis add that the same walking speed is achieved at a higher oxygen cost, that is, with reduced exercise efficiency (20, 21). Yet these exercise studies rarely assess the heart at the same time, rarely measure lung volume directly, and almost never report how often a patient who looks normal at rest is abnormal under load.

The practical consequence is that, standing in front of a patient before surgery, the clinician cannot answer three plain questions. How often does a normal resting result conceal a reduced reserve? Is there a structural measurement, ideally one that is independent of body size and that surgery can actually change, that predicts impairment better than the Cobb angle on which triage still leans, even though a Cobb threshold alone rarely uncovers meaningful cardiac findings (22)? Thoracic height is the natural candidate: it is the vertical, spine-determined component of thoracic volume, it is read from the standing radiograph obtained in every patient, it tracks lung volume in serial CT of early-onset scoliosis (23), and it is the dimension that growth-friendly and corrective surgery directly change (24), whereas rotation and rib-cage indices predict resting airflow but have not been linked to exercise reserve. And does the deformity predict the same thing for the static volume of the lungs as it does for the reserve drawn on during exertion? The systematic review that flagged the resting-to-exercise gap also named what was missing to close it: synchronous cardiac assessment, narrow age bands, stratification by etiology and sex, and an integrative exercise endpoint (19).

We designed this study to answer those questions within a single preoperative window. In consecutive adolescents with idiopathic or congenital scoliosis and in age- and sex-matched controls, we combined resting echocardiography, static pulmonary function testing, CT lung volume, and cardiopulmonary exercise testing, with three aims: to characterize the profile and magnitude of cardiopulmonary impairment; to quantify how far resting findings diverge from exercise findings in the same patient; and, using body-size-adjusted analysis, to identify the structural determinants of that impairment. Our central hypothesis was that deformity morphology governs the static volume of the thorax, captured by thoracic height and reducible to a usable threshold, but not its dynamic reserve, so that the two dimensions of preoperative cardiopulmonary risk have to be measured separately rather than inferred one from the other.

## Methods

### Study design and participants

This single-center case-control study was conducted at the Department of Orthopedics, the Second Affiliated Hospital of Kunming Medical University, between July 2023 and October 2025 (ethics approval PJ-2025-347; written informed consent obtained). Consecutive patients scheduled for scoliosis correction were screened, and their data were extracted retrospectively from the preoperative work-up, which routinely includes echocardiography, pulmonary function testing, chest CT and cardiopulmonary exercise testing before correction of severe deformity. Inclusion criteria were: (1) age 10–20 years; (2) clinically diagnosed AIS or CS with a coronal main-curve Cobb angle ≥ 10°; (3) standing whole-spine posteroanterior and lateral radiographs, supine side-bending radiographs, transthoracic echocardiography, static pulmonary function testing (PFT), chest CT, and cardiopulmonary exercise testing (CPET) completed within one month before surgery; and (4) complete, legible records. Exclusion criteria were secondary scoliosis from neuromuscular disease or syndromes, severe cardiopulmonary disease, prior thoracic or spinal surgery, and a CPET not reaching maximal effort [peak respiratory exchange ratio (RER) < 1.05]. The control group comprised 20 healthy children and adolescents without spinal deformity (negative Adam forward-bend test), drawn during the same period from healthy siblings or relatives accompanying patients to the scoliosis clinic, screen-negative peers attending the clinic for scoliosis screening, and healthy adolescents who volunteered for the study. Controls were frequency-matched to patients for age and sex and underwent the same echocardiography, PFT and CPET on the same equipment in the same laboratories. The final sample comprised 53 participants: 33 patients (21 AIS, 12 CS) and 20 controls.

### Radiographic and deformity assessment

A senior spine surgeon blinded to the functional results measured all parameters in triplicate on standing whole-spine radiographs and averaged them: main-curve Cobb angle and thoracic height (vertical distance between the T1 and T12 vertebral centroids, cm) on the posteroanterior film; thoracic kyphosis and lumbar lordosis on the lateral film; curve flexibility, calculated as (standing Cobb−side-bending Cobb)/standing Cobb   ×   100%, from the supine side-bending films; and apical rotation (Nash-Moe). Patients were stratified by Cobb angle into mild (< 45°, *n* = 5), moderate (45–90°, *n* = 22), and severe (> 90°, *n* = 6) subgroups.

### Multimodal cardiopulmonary assessment

Echocardiography (Vivid; performed and interpreted according to American Society of Echocardiography and European Association of Cardiovascular Imaging recommendations (25) yielded left ventricular ejection fraction (LVEF, biplane Simpson), fractional shortening, stroke volume, cardiac output and pulmonary artery systolic pressure; the device-reported cardiac index was not used because the height and weight entered at the console were self-reported. Static spirometry (MasterScreen, Jaeger/Vyaire) recorded FVC, FEV₁, VC max and MEF25/50/75. Because standing height is shortened by the coronal deformity, arm span was measured in all patients and entered as height for the derivation of predicted values, in line with ATS/ERS recommendations (26); for controls standing height was used (arm span and height differed by ≤ 4 cm). Percent-predicted values and z-scores for FVC, FEV₁ and FEV₁/FVC were recalculated from the measured volumes with the race-neutral GLI Global 2022 equations (27), which cover the 3–95-year range with a single continuous model and therefore avoid the discontinuity between paediatric and adult equations at 18 years; restriction was defined as FVC below the lower limit of normal (LLN, z < −1.645) (28) or, for comparability with previous studies, FVC < 80% predicted. Sensitivity analyses used the Chinese reference equations of Jian et al. (29) and the device-default equations (Zapletal for < 18 years, ECCS for ≥ 18 years) (30, 31), which reproduce the values printed on the original reports. Because no GLI equation exists for VC max, VC max is reported in litres and its %predicted (device equations) only as a supplementary index; MEF %predicted values are those of the device. CT lung volume was reconstructed in three dimensions from chest DICOM data by two independent spine surgeons, using the mean of the two measurements (inter-measurement reliability reported below). CPET was performed on an electronically braked cycle ergometer (Schiller CS-200 Ergospiro) with a symptom-limited ramp protocol (3 min rest, 3 min unloaded pedalling, individualized ramp to exhaustion in 8–12 min, 5 min recovery) according to ATS/ACCP recommendations (32). Breath-by-breath data yielded VO₂peak, anaerobic threshold (AT, V-slope method (33), VE/VCO₂ slope, METs, peak heart rate, peak RER, peak work rate, and the Weber and ventilatory classes. Peak effort required RER  ≥ 1.05; all 33 patients and all 20 controls met this criterion (RER ≥ 1.10 in 26 and 20, respectively). The reason for termination was recorded: leg fatigue in 31 of 33 patients (with RER ≥ 1.15 also reached in 3) and 19 of 20 controls, RER ≥ 1.15 alone in one patient and one control, and exhaustion of ventilatory reserve with oxygen desaturation in one patient with a 153° curve. No test was stopped for chest pain, arrhythmia or an abnormal blood-pressure response. VO₂peak %predicted is the device-reported value and is presented for description only; group comparisons of exercise capacity used weight-normalized values against the matched controls.

Derived variables. Body surface area was calculated by the Mosteller formula (34) from arm span (patients) or standing height (controls) and measured weight; cardiac index = cardiac output ÷ BSA; BMI = weight ÷ standing height^2^; VO₂peak and AT were normalized to body weight; FEV₁/FVC was computed from measured values; O₂ pulse = VO₂peak ÷ peak heart rate, expressed also as a percentage of the device-predicted VO₂ ÷ predicted heart rate; peak work rate was normalized to weight; curve flexibility = (standing Cobb−bending Cobb)/standing Cobb × 100.

### Statistical analysis

Analyses used SPSS 26.0 and Python 3.11, with normality checked by the Shapiro–Wilk test. Two-group comparisons used the Welch t or Mann–Whitney U test with Hedges’ g, and within each physiological domain (cardiac, pulmonary, CPET) *p* values were adjusted by the Benjamini-Hochberg false discovery rate (q). Severity subgroups were compared by ANOVA or Kruskal–Wallis, CS versus AIS by ANCOVA adjusting for thoracic height, age and sex (with Cobb-angle adjustment as a sensitivity analysis), and associations by Spearman correlation. Independent determinants were examined by hierarchical multivariable linear regression on percent-predicted or weight-normalized outcomes, with absolute CT lung volume additionally adjusted for height and weight. Each model was built on an age and sex base, and the incremental adjusted R^2^ (*Δ*R^2^) of adding the Cobb angle, thoracic height, or kyphosis was computed; nested models (base + Cobb + kyphosis, with versus without thoracic height) were compared by partial *F*-test and *Δ*AIC, reporting the unstandardized B (per cm) with 95% CI, standardized *β*, and variance inflation factor (VIF). Deformity predictors of FVC %predicted were screened by LASSO with 5-fold cross-validation. A resting–exercise mismatch cross-tabulated resting-normal patients (LVEF ≥ 55% and FVC ≥ 80% predicted; secondarily FVC ≥ LLN) against an abnormal exercise response (Weber ≥ B or VO₂peak < 80% predicted). Thoracic-height discrimination was assessed by ROC analysis with the Youden cutoff and 5-fold cross-validated AUC, and CT-volume reliability by the inter-measurement coefficient of variation. Sensitivity analyses repeated the main comparisons (i) restricting patients to peak RER ≥ 1.10, (ii) with the Jian and device-default reference equations, and (iii) with Du Bois and standing-height BSA for cardiac index. A two-sided *p* < 0.05 was considered significant.

## Results

### Baseline characteristics

Patients (*n* = 33; 21 AIS, 12 CS; 24 female) and controls (*n* = 20; 12 female) were comparable in age (14.5 ± 2.5 vs. 15.6 ± 3.1 years, *p* = 0.19), sex (*p* = 0.51) and BMI (*p* = 0.18), but patients were shorter (152.0 ± 12.7 vs. 163.7 ± 11.3 cm, *p* = 0.001), had a shorter arm span (154.1 ± 12.6 vs. 162.2 ± 11.4 cm, *p* = 0.020), were lighter (45.1 ± 10.7 vs. 55.7 ± 12.4 kg, *p* = 0.003), and had a smaller body surface area (1.38 ± 0.21 vs. 1.59 ± 0.23 m^2^, *p* = 0.003) (Table 1). In patients, arm span exceeded standing height by a mean of 2.1 cm and by up to 25 cm in the most severe curves. Mean Cobb angle was 72.5 ± 29.6° (range 27–153°), thoracic height 20.8 ± 3.8 cm, and thoracic kyphosis 32.5 ± 25.8°. Inter-measurement reliability of CT lung volume was excellent (coefficient of variation 1.7%). All participants reached a peak RER ≥ 1.05, so no patient was excluded for submaximal effort.

**Table 1:** Baseline characteristics.

Variable	Control (*n* = 20)	Scoliosis (*n* = 33)	P
Age, years	15.6 ± 3.1	14.5 ± 2.5	0.19
Sex, F/M	12/8	24/9	0.51
Height, cm	163.7 ± 11.3	152.0 ± 12.7	0.001
Arm span, cm	162.2 ± 11.4	154.1 ± 12.6	0.020
Weight, kg	55.7 ± 12.4	45.1 ± 10.7	0.003
BMI, kg/m^2^	20.5 ± 2.6	19.4 ± 3.6	0.18
BSA (Mosteller), m^2^	1.59 ± 0.23	1.38 ± 0.21	0.003

### Resting indices preserved, exercise reserve reduced

Resting cardiac parameters did not differ (LVEF 66.3 ± 4.2 vs. 65.5 ± 5.8%, fractional shortening, cardiac output 4.82 ± 0.68 vs. 4.96 ± 0.49 L/min, stroke volume and pulmonary artery systolic pressure, all q ≥ 0.84); cardiac index was, if anything, higher in patients   (3.54 ± 0.54 vs. 3.19 ± 0.58 L/min/m^2^, q = 0.12), a pattern unchanged with Du Bois  or standing-height BSA (Supplementary Table S1). Spirometry showed a restrictive pattern: FVC %predicted (72.0 ± 28.1 vs. 92.8 ± 10.0; q < 0.001, g = 0.89; z-score −2.17 ± 2.16 vs. −0.54 ± 0.75) and FEV₁ %predicted (70.3 ± 28.3 vs. 88.4 ± 10.7; q = 0.002) were reduced, FVC fell below the LLN in 18 of 33 patients and 1 of 20 controls, FEV₁/FVC did not differ (86.5 ± 8.5 vs. 83.7 ± 6.2%, q = 0.13), and all MEF flows   were reduced (q < 0.001). On CPET, VO₂peak (0.98 ± 0.34 vs. 1.66 ± 0.60 L/min; 21.8 ± 6.2 vs. 29.6 ± 6.2 mL/min/kg; q < 0.001, g = 1.24), AT (14.2 ± 3.4 vs. 18.9 ± 5.0 mL/min/kg, q = 0.001), METs, and peak work rate (84 ± 31 vs. 139 ± 48 W; 1.85 ± 0.53 vs. 2.49 ± 0.55 W/kg, q < 0.001) were   reduced, and the VE/VCO₂ slope was higher (32.4 ± 4.5 vs. 27.5 ± 1.5; q < 0.001). O₂ pulse was reduced (5.9 ± 1.9 vs. 9.7 ± 3.5 mL/beat; q < 0.001, g = 1.45; 72.8 ± 22.6 vs. 90.4 ± 17.0% predicted, q = 0.003) and fell below 80% predicted in 22 of 33 patients versus 4 of 20 controls. Peak heart rate (82 ± 8 vs. 85 ± 5% predicted) and peak RER (1.16 ± 0.08 vs. 1.17 ± 0.02) did not differ, indicating comparable effort. Ten of 33 patients (30.3%) were Weber class ≥ B (Table 2, Figure 1).

**Table 2:** Cardiopulmonary indices (mean ± SD; q = FDR-adjusted within domain; g = hedges’ g, control minus scoliosis).

Index	Control	Scoliosis	q (FDR)	Hedges g
LVEF, %	65.5 ± 5.8	66.3 ± 4.2	0.88	−0.17
Fractional shortening, %	36.6 ± 4.3	36.2 ± 4.4	0.96	0.07
Stroke volume, mL	66.1 ± 6.0	63.6 ± 15.1	0.84	0.19
Cardiac output, L/min	4.96 ± 0.49	4.82 ± 0.68	0.84	0.21
Cardiac index (CO/BSA), L/min/m^2^	3.19 ± 0.58	3.54 ± 0.54	0.12	−0.62
PASP, mmHg	27.7 ± 4.7	28.0 ± 5.7	0.96	−0.06
FVC, L	3.33 ± 0.71	2.17 ± 0.96	<0.001	1.31
FVC %pred (GLI)	92.8 ± 10.0	72.0 ± 28.1	<0.001	0.89
FVC z-score (GLI)	−0.54 ± 0.75	−2.17 ± 2.16	<0.001	0.90
FEV₁, L	2.76 ± 0.49	1.89 ± 0.85	<0.001	1.17
FEV₁ %pred (GLI)	88.4 ± 10.7	70.3 ± 28.3	0.002	0.76
FEV₁/FVC, %	83.7 ± 6.2	86.5 ± 8.5	0.13	−0.35
VC max, L	3.52 ± 0.74	2.40 ± 0.99	<0.001	1.22
MEF50%pred	100.0 ± 10.6	70.7 ± 30.7	<0.001	1.15
VO₂peak, L/min	1.66 ± 0.60	0.98 ± 0.34	<0.001	1.48
VO₂peak %pred (device)	76.5 ± 13.9	59.6 ± 17.7	<0.001	1.02
VO₂peak, mL/min/kg	29.6 ± 6.2	21.8 ± 6.2	<0.001	1.24
AT, mL/min/kg	18.9 ± 5.0	14.2 ± 3.4	0.001	1.13
METs	5.2 ± 1.5	4.0 ± 1.1	0.010	0.96
VE/VCO₂ slope	27.5 ± 1.5	32.4 ± 4.5	<0.001	−1.31
O₂ pulse, mL/beat	9.7 ± 3.5	5.9 ± 1.9	<0.001	1.45
O₂ pulse %pred	90.4 ± 17.0	72.8 ± 22.6	0.003	0.84
Peak heart rate %pred	84.8 ± 4.6	82.5 ± 7.8	0.18	0.34
Peak RER	1.17 ± 0.02	1.16 ± 0.08	0.15	0.18
Peak work rate, W	139 ± 48	84 ± 31	<0.001	1.43
Peak work rate, W/kg	2.49 ± 0.55	1.85 ± 0.53	<0.001	1.17

**Figure 1:**
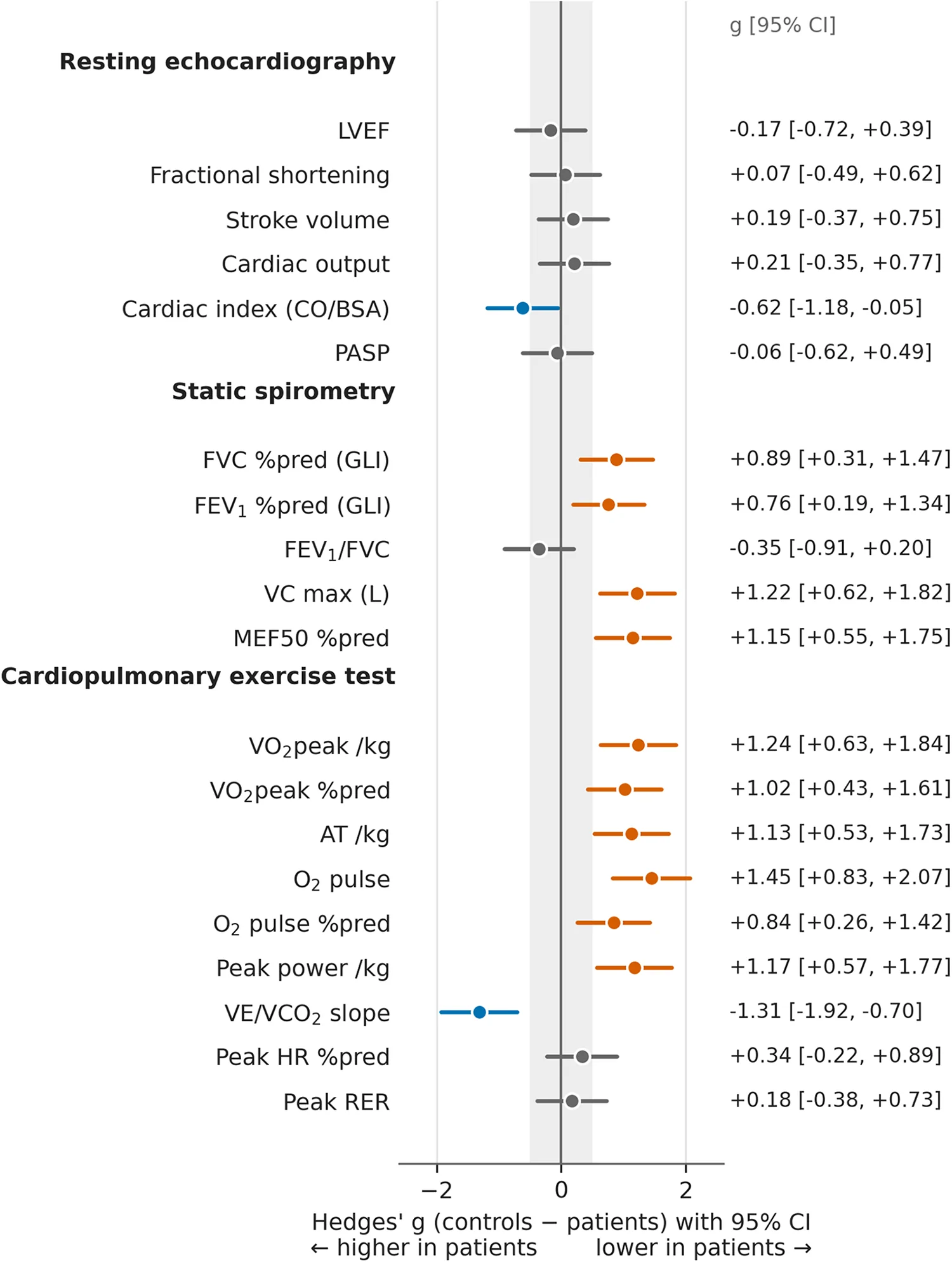
Effect sizes (hedges’ g, controls minus patients, with 95% CI) of resting echocardiographic, spirometric and CPET indices; positive values denote lower values in patients, and the shaded band marks |g| < 0.5.

### Resting–exercise mismatch

Thirteen patients were resting-normal (LVEF ≥ 55% and FVC ≥ 80% predicted), of whom 10 (76.9%) had an abnormal exercise response; with FVC ≥ LLN as the resting criterion, 12 of 15 (80.0%) were abnormal on exercise, and restricting to peak RER ≥ 1.10, 8 of 11 (72.7%) (Supplementary Tables S2, S3). Resting assessment thus missed a reduced reserve in roughly three-quarters of normal-appearing patients.

### Deformity severity

Across the mild, moderate and severe (> 90°) subgroups (*n* = 5, 22 and 6; ANOVA or Kruskal–Wallis as appropriate), pulmonary indices declined sharply in the severe subgroup: FVC %predicted 77.9/80.1/37.3 (ANOVA, *p* =   0.002) and MEF50%predicted (Kruskal–Wallis, *p* = 0.023), while VO₂peak/kg fell (25.7/22.3/16.6 mL/min/kg; ANOVA, *p* = 0.032) and the VE/VCO₂ slope rose (30.6/31.9/35.7; Kruskal–Wallis, *p* = 0.050); with six patients in the severe subgroup  these contrasts are descriptive.

### Independent determinants: thoracic height predicts restrictive lung volume, not exercise capacity

Thoracic height correlated with FVC %predicted (*ρ* = 0.72) and the Cobb angle correlated negatively (*ρ* = −0.61). In hierarchical regression adjusted for age and sex, thoracic height produced the largest incremental gain for restrictive lung-volume outcomes (*Δ* adjusted R^2^ = 0.47 for FVC %predicted, 0.53 for FEV₁ %predicted, 0.43 for VC max %predicted) and remained independent after further adjustment for the Cobb angle and kyphosis (FVC %predicted: B = 3.65 per cm, 95% CI 1.64–5.65, standardized *β* = 0.50, partial F = 13.9, *p* < 0.001, *Δ*AIC = −11.7; FEV₁ %predicted: B = 3.86, partial F = 16.5, *p* < 0.001; VC max %predicted: B = 3.24, partial F = 11.1, *p* = 0.003), with the Cobb angle also contributing (*p* = 0.004–0.008) and low collinearity (VIF ≤ 2.0). LASSO ranked thoracic height (standardized coefficient  + 14.5) and the Cobb angle (−12.8) far above the remaining deformity parameters. By contrast, after body-size adjustment no deformity parameter independently predicted exercise capacity (thoracic height for VO₂peak %predicted, VO₂peak/kg, AT/kg and O₂ pulse %predicted: *p*=0.30–0.80), and thoracic height added nothing to CT lung volume once height and weight were in the model (*p* = 0.64) (Table 3, Figure 2). Sex modified the lung-volume–deformity relationship (sex   ×   Cobb and sex   ×   thoracic height for FVC %predicted, *p* = 0.015 and 0.041) but not the exercise associations.

**Table 3:** Hierarchical regression (*n* = 33): thoracic height independently predicts lung-volume %predicted but not exercise capacity.

Outcome	+Cobb *Δ*R^2^	+Th.height *Δ*R^2^	Th.height B (95% CI)	partial F/p	*Δ*AIC
FVC %pred	0.32	0.47	3.65 (1.64–5.65)	13.9/ < 0.001	−11.7
FEV₁ %pred	0.35	0.53	3.86 (1.91–5.81)	16.5/ < 0.001	−13.7
VC max %pred	0.33	0.43	3.24 (1.25–5.24)	11.1/0.003	−9.4
VO₂peak %pred	0.06	0.05	0.62 (−1.25–2.48)	0.5/0.50	+1.4
VO₂peak/kg	0.16	0.11	0.28 (−0.26–0.82)	1.1/0.30	+0.7
AT/kg	−0.02	−0.01	0.03 (−0.23–0.30)	0.1/0.80	+1.9
O₂ pulse %pred	0.03	0.01	0.39 (−1.91–2.70)	0.1/0.73	+1.9

**Figure 2:**
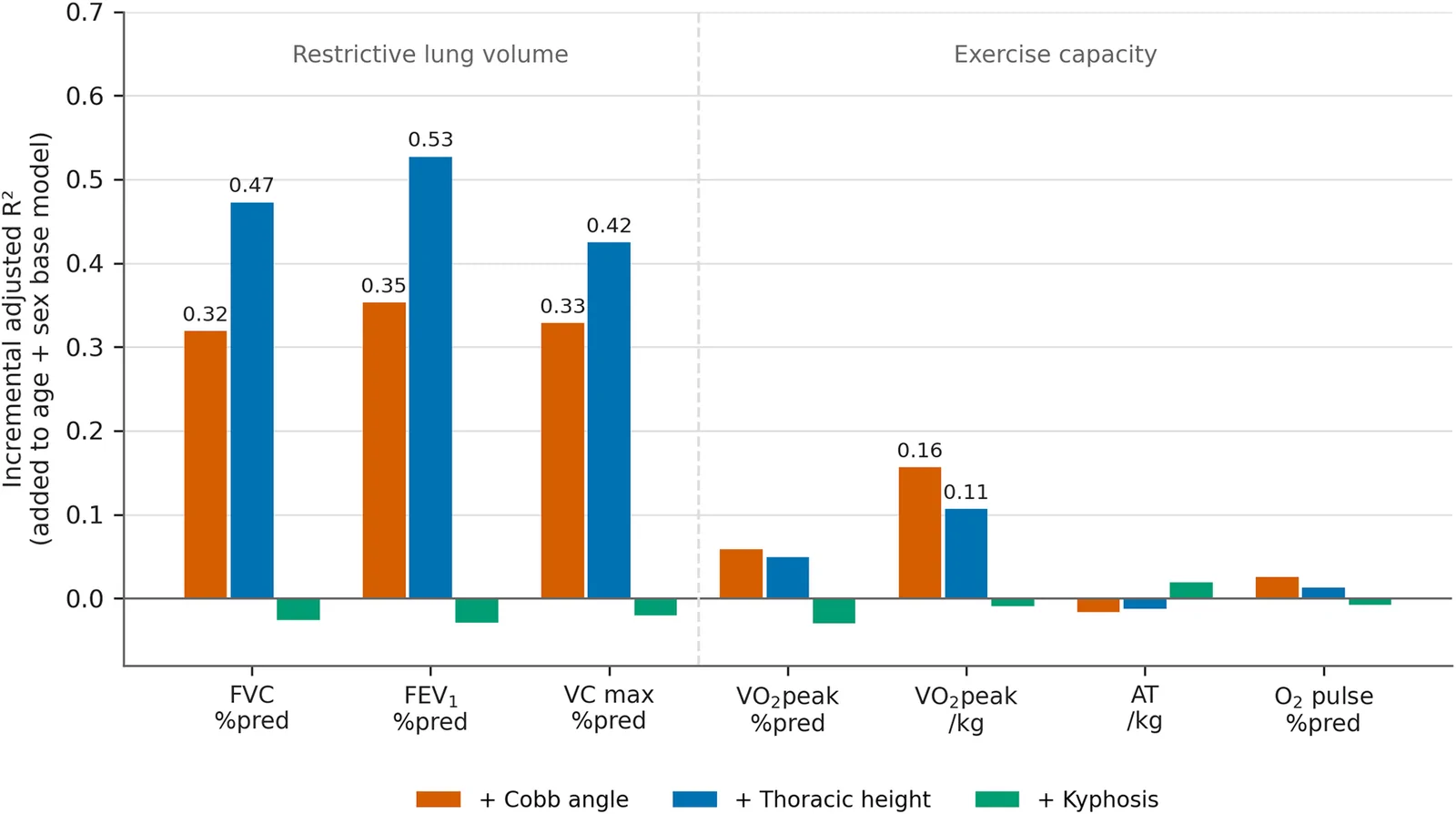
Incremental adjusted R^2^ of the cobb angle, thoracic height and kyphosis, each added to an age- and sex-adjusted base model, for lung-volume (left) and exercise (right) outcomes.

### Thoracic-height threshold for restrictive impairment

Thoracic height discriminated restrictive impairment and was stable on cross-validation: FVC < 80% predicted (AUC 0.90, 5-fold cross-validated 0.84; cutoff 19.6 cm; sensitivity 0.70, specificity 1.00), FVC < LLN (AUC 0.92, cross-validated 0.89; same cutoff) and VC max < 80% predicted (AUC 0.87, cross-validated 0.81). Discrimination for exercise endpoints was weaker and unstable (VO₂peak < 80% predicted: AUC 0.70, cross-validated 0.52; O₂ pulse < 80% predicted: AUC 0.65, cross-validated 0.55), consistent with the regression (Figure 3).

**Figure 3:**
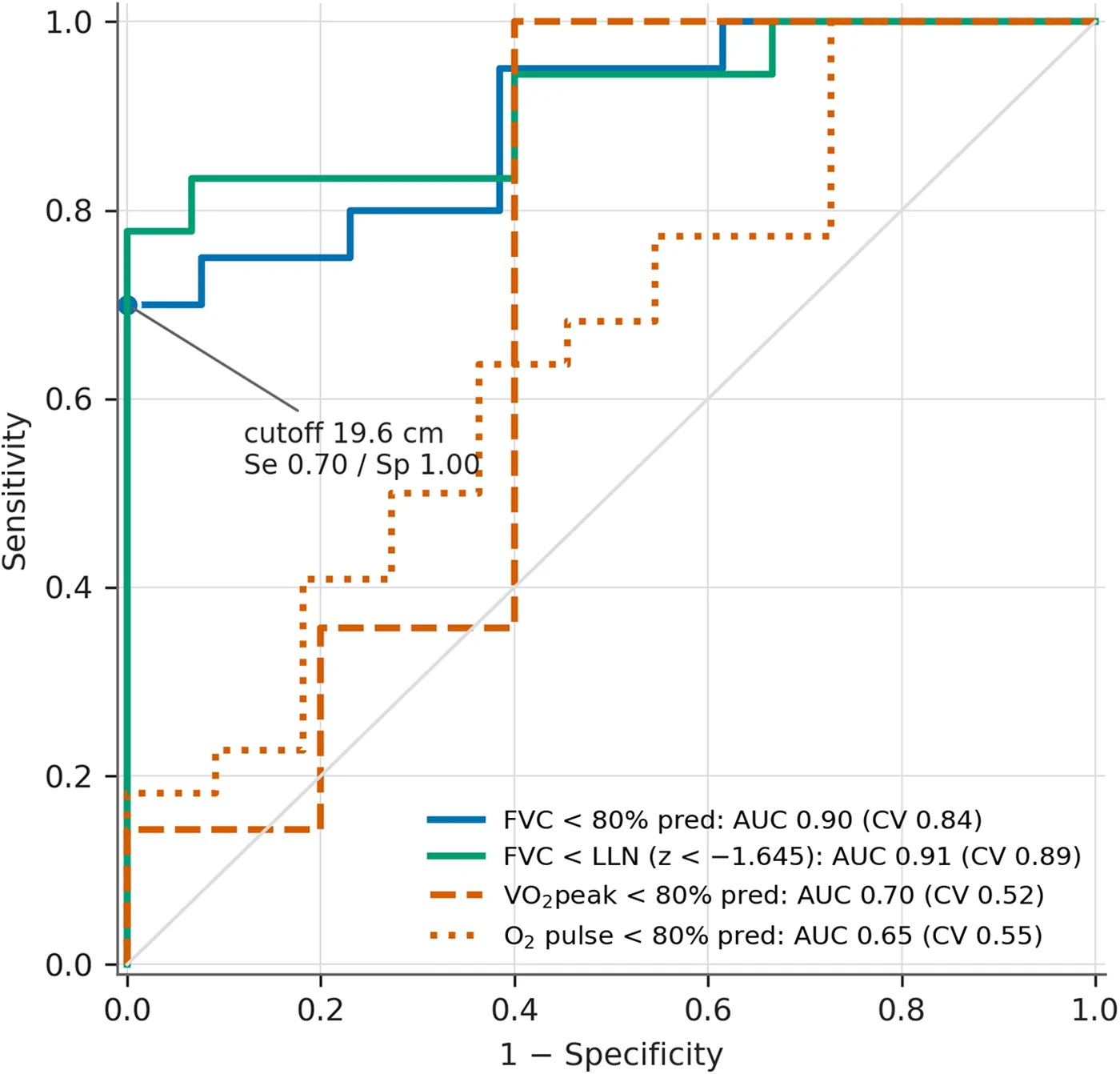
ROC curves of thoracic height for restrictive impairment (FVC < 80% predicted; FVC < LLN) and for reduced exercise capacity (VO₂peak and O₂ pulse < 80% predicted), with apparent and 5-fold cross-validated AUC; the marker shows the youden cutoff of 19.6 cm.

### Etiology: congenital versus idiopathic scoliosis

After adjustment for thoracic height, age and sex, CS had lower CT lung volume than AIS (adjusted means 1,881 vs. 2,356 cm^3^; *p* = 0.030), whereas FVC %predicted (69.0 vs. 73.7; *p* = 0.55), FEV₁ %predicted, VC max %predicted, MEF50%predicted (*p* = 0.083), and cardiac and CPET indices did not differ. With Cobb-angle adjustment, as in the original analysis, CS additionally showed lower MEF50%predicted (*p* = 0.008) and a trend towards lower FVC %predicted (*p* = 0.063) (Table 4, Supplementary Table S4): the etiological difference in static spirometry is accounted for by thoracic height, while the difference in CT lung volume is not.

**Table 4:** Congenital vs. idiopathic scoliosis, ANCOVA-adjusted for thoracic height, age and sex (Cobb-adjusted *p* for comparison).

Index	AIS	CS	*p* (thoracic height, age, sex)	*p* (Cobb-adjusted)
CT lung volume, cm^3^	2,356	1,881	0.030	0.013
FVC %pred	73.7	69.0	0.55	0.063
FEV₁ %pred	72.1	67.2	0.53	0.063
VC max %pred	78.3	74.7	0.64	0.13
MEF50%pred	76.6	60.2	0.083	0.008
LVEF, %	66.0	66.8	0.60	0.73
Cardiac output, L/min	4.9	4.6	0.20	0.084
VO₂peak, mL/min/kg	21.1	23.0	0.32	0.20
AT, mL/min/kg	14.2	14.2	0.99	0.30
O₂ pulse %pred	71.9	74.3	0.78	0.85

## Discussion

We assessed resting echocardiography, static pulmonary function, CT lung volume, and cardiopulmonary exercise testing in the same preoperative scoliosis patients and in matched controls, and tested which deformity parameters predict lung volume and which predict exercise capacity. Resting cardiac function and much of resting spirometry were preserved, whereas exercise capacity and ventilatory efficiency were markedly reduced, O₂ pulse was low despite normal resting echocardiography, and most patients who were normal at rest were already abnormal on exercise. After adjustment for body size, thoracic height predicted restrictive lung volume, while no single deformity parameter predicted exercise reserve.

The most consequential cardiopulmonary deficit in these patients is the one the standard preoperative work-up is structurally unable to detect. Resting cardiac function and much of resting spirometry were preserved, yet peak oxygen uptake and the anaerobic threshold were reduced by roughly a quarter per kilogram and by 40% in absolute terms, and ventilatory efficiency was degraded; decisively, the discordance held at the level of the individual, with three-quarters of the patients who met resting-normal criteria (10 of 13) already abnormal on exercise. This is not measurement scatter but a predictable consequence of loading: a stiffened, deformed chest exacts a ventilatory penalty that materializes only as metabolic demand rises, so resting spirometry and echocardiography interrogate the physiology at the one operating point where it still looks intact (2). A recent systematic review reached the same conclusion qualitatively and identified VO₂max as the most integrative marker of physiological burden (19); our data give that discordance a magnitude. The implication is not that resting tests are wrong but that they are aimed at the wrong target: a pathway anchored on resting normality and a Cobb-angle referral trigger is calibrated to the least impaired component while remaining blind to the one that most plausibly governs perioperative tolerance. Preoperative assessment therefore has to provoke reserve and measure it, not certify its presence at rest.

The cardiac component of this mismatch deserves emphasis. Resting echocardiography was normal, consistent with a series of 294 patients with idiopathic scoliosis in which resting chamber dimensions and ejection fraction fell only beyond 90° and cardiac index only beyond 120° (35); the mean curve here was 72°. Yet O₂ pulse, the product of stroke volume and arteriovenous oxygen difference, was reduced by 40% and fell below 80% of predicted in two-thirds of patients, at a peak heart rate and RER no different from controls. Because peripheral oxygen extraction is not impaired in scoliosis, a low O₂ pulse at comparable effort implies a limited stroke-volume response to exercise, an exercise cardiac limitation that resting imaging cannot show. Preoperative echocardiography retains a distinct role, since in surgical cohorts of idiopathic scoliosis it detects congenital heart disease in about 4% and valvular abnormalities in about 30% (36); its purpose is the screening of structural disease, whereas functional reserve has to be provoked. Exercise echocardiography and right-ventricular strain imaging, which were not performed here, would be the direct way to characterize the mechanism.

Because the deformity shortens standing height, predicted volumes derived from it are underestimated and percent-predicted values correspondingly inflated. We therefore derived predicted values from arm span, which exceeded standing height by up to 25 cm in the most severe curves, and used a single continuous reference equation across the age range. The restrictive pattern and the 19.6-cm thoracic-height threshold were unchanged with alternative equations or with restriction defined by the lower limit of normal (Supplementary Table S5).

The structural geometry of the deformity governs the static volume of the thorax, and thoracic height is the parameter that captures it. After adjustment for age, sex, Cobb angle and kyphosis, thoracic height independently predicted restrictive lung-volume impairment (FVC %predicted B = 3.65 per cm, 95% CI 1.64–5.65, partial F = 13.9, *Δ*AIC = −11.7; VC max %predicted partial F = 11.1), with low collinearity (VIF ≤ 2.0) and a cross-validated discrimination threshold of 19.6 cm. The reason it, rather than curve magnitude, carries this signal is anatomical: thoracic volume is set by the width and depth of the rib cage together with the height the thoracic spine provides, whereas the capacity to change that volume depends on respiratory-muscle function (37). Thoracic height is the vertical, spine-determined component of that volume. Serial-CT data in early-onset scoliosis support the same hierarchy, with T1–T12 length tracking lung volume while curve magnitude does not (23). This places our result alongside, not against, the report that a 2D Cobb angle outperforms a predicted-3D kyphosis for FEV₁ (13), because that comparison concerns an airflow ratio and contrasts the Cobb angle with a sagittal-curvature surrogate, whereas ours concerns lung volume and contrasts deformity with vertical growth. It also refines the early-onset 18-cm threshold, which a later reassessment found unreliable as a single outcome target (24), by establishing a body-size-independent, cross-validated threshold for restrictive volume in the preoperative AIS and CS population, where it had not been tested.

What governs the volume of the thorax does not govern its reserve, and the contrast was quantitative rather than merely directional. Thoracic height added substantial explanatory value for restrictive volume (incremental adjusted R^2^ 0.47 for FVC %predicted, 0.53 for FEV₁ %predicted and 0.43 for VC max %predicted) but essentially none for exercise endpoints, where neither thoracic height nor the Cobb angle nor kyphosis independently predicted peak oxygen uptake, the anaerobic threshold or O₂ pulse after body-size adjustment (all *p* ≥ 0.30). Discrimination behaved the same way: thoracic height separated restrictive lung-volume impairment well and stably (AUC 0.90, cross-validated 0.84) but discriminated a reduced VO₂peak only weakly and unstably (AUC 0.70, cross-validated 0.52). This dissociation is the second half of the same anatomical logic: the capacity to change thoracic volume, as distinct from the volume itself, depends on the diaphragm and the secondary muscles of respiration (37), and that dynamic function is not legible in any static measure of vertical growth. The deficit left unexplained is large rather than marginal, with exercise capacity, O₂ pulse and ventilatory efficiency impaired at effect sizes of 1.1 to 1.5, so exercise reserve integrates contributions no radiograph contains: the elevated elastic work of a stiff chest wall, ventilatory inefficiency, a limited stroke-volume response, and peripheral deconditioning. This reconciles our data with cohorts reporting that the aerobic deficit in scoliosis is largely independent of the Cobb angle (15, 16) and extends them by showing the independence holds against thoracic height and kyphosis as well.

Etiology acted on the volume axis alone. At matched thoracic height, congenital scoliosis retained a lower CT lung volume than idiopathic scoliosis (1,881 vs. 2,356 cm^3^), whereas  the differences in static spirometry seen after Cobb-angle adjustment disappeared once thoracic height was accounted for. The two findings are complementary: the spirometric penalty of a congenital curve is largely the penalty of a shorter thorax, but the parenchymal volume penalty is not. We interpret this as the difference between mechanical and developmental restriction. In AIS the deformity compresses a lung whose alveolar complement is essentially complete, so restriction scales with the space   available and is captured by thoracic height. In    CS the vertebral and rib malformations constrain the thorax during the first years of life, when alveolar multiplication is still proceeding (37, 38); in a porcine model of early-onset scoliosis with thoracic insufficiency, early tethering of the spine and ribs reduced total lung volume and the number and size of alveoli, with collagen deposition in the lung (39), and the steeper pulmonary decline reported in congenital scoliosis points the same way (18). A smaller lung for a given thoracic height is what a developmental deficit would look like. The hypothesis is testable, by diffusing capacity, CT lung density and stratification by age at onset, and the subgroup (*n* = 12) is too small to do more than propose it.

Taken together, these findings argue for augmenting rather than replacing the conventional preoperative work-up, and they specify how. Thoracic height has three properties that make it useful for surgical planning rather than for description alone. It is body-size independent, so it flags restrictive risk that absolute volumes confound with a short or light patient; it carries a cross-validated threshold near 20 cm, giving a concrete decision point rather than a continuous correlation; and it measures a quantity that surgery changes. Corrective and growth-friendly constructs lengthen the instrumented thoracic spine, and serial CT in growing patients shows that gains in T1–T12 length track gains in lung volume, whereas curve magnitude and its correction do not (14, 23). In practice, a thoracic height below about 20 cm on the preoperative radiograph identifies the patient whose restriction is volume-limited: in the growing child this favours a growth-friendly strategy that lengthens the thoracic spine, whereas in the skeletally mature patient it argues for a correction that maximizes thoracic height and for restrictive-risk counselling before definitive fusion. The threshold is derived from 33 patients and needs prospective validation before it is used as a decision rule.

Exercise reserve integrates what the resting tests measure separately. The ventilatory penalty of a stiff, asymmetric thorax, the metabolic cost of a less efficient breathing pattern and the stroke-volume response of the heart are summed under load into a single quantity, peak oxygen uptake, and its determinants can be read from the accompanying variables. In our patients the deficit was of the same order as in the larger cycle-ergometer cohorts of idiopathic and congenital scoliosis (16, 18) and in the pooled estimate of a recent systematic review (19), and it appeared together with a raised VE/VCO₂ slope and a reduced O₂ pulse, the two signatures of ventilatory inefficiency and of a limited stroke-volume response. The pattern differs from that of children after growth-sparing surgery for early-onset scoliosis, who keep pace with their peers at a higher oxygen cost per unit of work (20, 21): in the untreated adolescent thorax the limitation is one of capacity, not only of efficiency. Respiratory events are the commonest major non-neurological complication of deformity correction, in 12%–14% of vertebral column resections (3, 40) and 22% of growth-friendly procedures (4), with non-idiopathic etiology and low FVC as their established risk factors; a measure of reserve that is already reduced in three-quarters of resting-normal patients is the natural candidate to sharpen that stratification, and its incremental value over FVC and etiology is the object of a prospective study now under way. In the meantime, reduced reserve identifies the patients in whom inspiratory-muscle training before surgery has been shown to improve lung function and functional capacity (41).

These two findings organize preoperative care along the axes they define. For surgical strategy, thoracic height adds a volume-protection consideration to correction planning and to the choice and timing of growth-friendly versus definitive constructs. For preoperative management, a short thoracic height on the radiograph and a low reserve on CPET select, respectively, the patients who need restrictive-risk counselling and those who need formal cardiopulmonary risk stratification, in place of a single Cobb-angle trigger that rarely changes management (22). For postoperative rehabilitation, because corrective surgery preserves rather than restores cardiopulmonary capacity (19), the low-reserve patients identified beforehand are the rational targets for inspiratory-muscle and aerobic prehabilitation (41). The static axis is read from a radiograph already obtained in every patient; the dynamic axis requires one added test. Neither replaces the other, and together they give a patient-tailored preoperative picture that the Cobb angle and spirometry do not.

The study's main strength is a four-modality cardiopulmonary assessment with matched controls and body-size-adjusted, cross-validated statistics. Several limitations affect interpretation. The sample is small and single-center (33 patients, with severe *n* = 6, congenital *n* = 12 and resting-normal *n* = 13 subgroups), so the resting–exercise mismatch rate and the thoracic-height threshold are preliminary and need external validation. Cardiac function was assessed by resting echocardiography only; without exercise echocardiography or right-ventricular strain imaging, the mechanism of the reduced O₂ pulse, whether stroke-volume limitation, right-heart strain or both, cannot be resolved, and this is the principal target for a prospective exercise-imaging study. VO₂peak %predicted relied on device-default reference equations, so inferences were based on weight-normalized values against matched controls. Finally, controls were a convenience sample of healthy relatives, screen-negative peers and volunteers rather than a population-based sample, and one control fell below the LLN for FVC.

## Conclusion

In preoperative scoliosis, resting cardiac function and much of resting pulmonary function are preserved, yet exercise reserve is frequently and occultly reduced, missed by resting assessment in roughly three-quarters of normal-appearing patients. Deformity morphology governs the static volume of the thorax but not its dynamic reserve: thoracic height is a body-size-independent predictor of restrictive lung volume with a clinically usable threshold near 20 cm, whereas exercise reserve is multifactorial, includes an exercise cardiac limitation revealed by O₂ pulse, and must be measured directly by CPET. Integrating thoracic-height-based stratification and CPET into preoperative evaluation addresses two distinct dimensions of cardiopulmonary risk that neither the Cobb angle nor spirometry captures alone.

## Data Availability

The original contributions presented in the study are included in the article/Supplementary Material, further inquiries can be directed to the corresponding authors.
