# Supplementary material for: Comparison of cardiopulmonary function in patients with scoliosis at rest and during exercise: a case-control study
Source: Front Surg. 2026 Oct 13;13:1931071. doi: 10.3389/fsurg.2026.1931071 (PMC13710326; doi:10.3389/fsurg.2026.1931071)
Supplement: Supplementary file 1 [file Table1.docx]

# Supplementary Material

Comparison of Cardiopulmonary Function in Patients with Scoliosis at Rest and During Exercise: A Case-Control Study

## Supplementary Table S1. Cardiac index with alternative body-surface-area formulas

| **Index** | **Control (n=20)** | **Scoliosis (n=33)** | **p** | **Hedges g** |
| --- | --- | --- | --- | --- |
| Cardiac output, L/min | 4.96 ± 0.49 | 4.82 ± 0.68 | 0.420 | 0.21 |
| CI, Mosteller BSA (arm span in patients; primary) | 3.19 ± 0.58 | 3.54 ± 0.54 | 0.020 | -0.62 |
| CI, Du Bois BSA (arm span in patients) | 3.17 ± 0.57 | 3.51 ± 0.52 | 0.027 | -0.61 |
| CI, Mosteller BSA (standing height in all) | 3.19 ± 0.58 | 3.56 ± 0.55 | 0.014 | -0.65 |

## Supplementary Table S2. Resting-normal patients (LVEF ≥ 55% and FVC ≥ 80% predicted, n = 13) and their exercise response

| **Patient** | **Etiology** | **LVEF, %** | **FVC %pred** | **VO₂peak %pred** | **Weber class** | **O₂ pulse %pred** | **Abnormal exercise response** |
| --- | --- | --- | --- | --- | --- | --- | --- |
| P06 | AIS | 66 | 96.3 | 63 | A | 72.1 | Yes |
| P07 | CS | 61 | 91.3 | 81 | A | 94.9 | No |
| P09 | AIS | 62 | 88.7 | 93 | A | 106.1 | No |
| P10 | AIS | 72 | 108.4 | 41 | C | 54.8 | Yes |
| P17 | AIS | 68 | 82.2 | 62 | A | 72.2 | Yes |
| P19 | AIS | 70 | 105.5 | 72 | A | 79.7 | Yes |
| P23 | CS | 68 | 104.0 | 88 | A | 115.6 | No |
| P27 | CS | 70 | 94.9 | 70 | A | 85.3 | Yes |
| P28 | AIS | 64 | 97.3 | 64 | A-C | 72.4 | Yes |
| P29 | AIS | 72 | 98.7 | 67 | A | 88.4 | Yes |
| P31 | AIS | 73 | 89.5 | 59 | A | 72.9 | Yes |
| P32 | AIS | 68 | 95.5 | 69 | A | 87.9 | Yes |
| P33 | AIS | 69 | 144.4 | 34 | B | 34.2 | Yes |

Abnormal exercise response = Weber class ≥ B or VO₂peak < 80% predicted. Ten of 13 (76.9%) were abnormal; with FVC ≥ LLN as the resting criterion, 12 of 15 (80.0%).

## Supplementary Table S3. Sensitivity analysis restricting patients to peak RER ≥ 1.10 (26 patients vs 20 controls)

| **Index** | **Control (n=20)** | **Scoliosis (n=26)** | **q (FDR)** | **Hedges g** |
| --- | --- | --- | --- | --- |
| Cardiac index, L/min/m² | 3.19 ± 0.58 | 3.56 ± 0.50 | 0.106 | -0.67 |
| FVC %pred | 92.82 ± 10.02 | 72.97 ± 29.54 | 0.004 | 0.84 |
| VO₂peak, mL/min/kg | 29.55 ± 6.21 | 22.05 ± 6.60 | <0.001 | 1.15 |
| AT, mL/min/kg | 18.88 ± 5.03 | 14.15 ± 3.46 | 0.002 | 1.10 |
| VE/VCO₂ slope | 27.50 ± 1.46 | 32.70 ± 4.62 | <0.001 | -1.41 |
| O₂ pulse, mL/beat | 9.68 ± 3.47 | 5.84 ± 2.07 | <0.001 | 1.36 |
| O₂ pulse %pred | 90.44 ± 16.98 | 72.36 ± 20.78 | 0.003 | 0.92 |

| **Outcome** | **ΔR² thoracic height** | **B per cm (95% CI)** | **p** |
| --- | --- | --- | --- |
| FVC %pred | 0.58 | 3.76 (1.26–6.25) | 0.005 |
| FEV₁ %pred | 0.61 | 3.81 (1.35–6.27) | 0.004 |
| VC max %pred | 0.52 | 3.18 (0.70–5.67) | 0.015 |
| VO₂peak/kg | 0.15 | 0.45 (-0.18–1.08) | 0.152 |
| AT/kg | -0.01 | 0.04 (-0.28–0.36) | 0.806 |
| O₂ pulse %pred | 0.13 | 1.72 (-0.80–4.25) | 0.169 |

Resting–exercise mismatch in this subset: 8 of 11 resting-normal patients (72.7%).

## Supplementary Table S4. Congenital vs idiopathic scoliosis: ANCOVA with alternative covariate sets

| **Index** | **Model** | **AIS adjusted mean** | **CS adjusted mean** | **p** |
| --- | --- | --- | --- | --- |
| CT lung volume, cm³ | Thoracic height + age + sex (primary) | 2355.6 | 1880.9 | 0.030 |
| CT lung volume, cm³ | Cobb angle (original) | 2419.5 | 1769.1 | 0.013 |
| CT lung volume, cm³ | Thoracic height + Cobb + age + sex | 2365.8 | 1863.0 | 0.024 |
| FVC %pred | Thoracic height + age + sex (primary) | 73.7 | 69.0 | 0.545 |
| FVC %pred | Cobb angle (original) | 77.5 | 62.2 | 0.063 |
| FVC %pred | Thoracic height + Cobb + age + sex | 74.5 | 67.6 | 0.352 |
| FVC z-score | Thoracic height + age + sex (primary) | -2.0 | -2.4 | 0.550 |
| FVC z-score | Cobb angle (original) | -1.7 | -2.9 | 0.058 |
| FVC z-score | Thoracic height + Cobb + age + sex | -2.0 | -2.5 | 0.350 |
| FEV₁ %pred | Thoracic height + age + sex (primary) | 72.1 | 67.2 | 0.530 |
| FEV₁ %pred | Cobb angle (original) | 75.8 | 60.7 | 0.063 |
| FEV₁ %pred | Thoracic height + Cobb + age + sex | 72.9 | 65.8 | 0.328 |
| FEV₁/FVC, % | Thoracic height + age + sex (primary) | 86.6 | 86.4 | 0.947 |
| FEV₁/FVC, % | Cobb angle (original) | 86.5 | 86.4 | 0.971 |
| FEV₁/FVC, % | Thoracic height + Cobb + age + sex | 86.8 | 86.0 | 0.819 |
| MEF50 %pred | Thoracic height + age + sex (primary) | 76.6 | 60.2 | 0.083 |
| MEF50 %pred | Cobb angle (original) | 79.8 | 54.7 | 0.008 |
| MEF50 %pred | Thoracic height + Cobb + age + sex | 77.5 | 58.7 | 0.043 |
| VC max %pred | Thoracic height + age + sex (primary) | 78.3 | 74.7 | 0.644 |
| VC max %pred | Cobb angle (original) | 82.2 | 68.0 | 0.075 |
| VC max %pred | Thoracic height + Cobb + age + sex | 79.2 | 73.2 | 0.414 |
| LVEF, % | Thoracic height + age + sex (primary) | 66.0 | 66.8 | 0.595 |
| LVEF, % | Cobb angle (original) | 66.5 | 65.9 | 0.727 |
| LVEF, % | Thoracic height + Cobb + age + sex | 66.1 | 66.6 | 0.733 |
| Cardiac output, L/min | Thoracic height + age + sex (primary) | 4.9 | 4.6 | 0.202 |
| Cardiac output, L/min | Cobb angle (original) | 5.0 | 4.6 | 0.084 |
| Cardiac output, L/min | Thoracic height + Cobb + age + sex | 4.9 | 4.6 | 0.203 |
| Cardiac index, L/min/m² | Thoracic height + age + sex (primary) | 3.4 | 3.7 | 0.169 |
| Cardiac index, L/min/m² | Cobb angle (original) | 3.4 | 3.7 | 0.157 |
| Cardiac index, L/min/m² | Thoracic height + Cobb + age + sex | 3.5 | 3.7 | 0.218 |
| VO₂peak, L/min | Thoracic height + age + sex (primary) | 1.0 | 0.9 | 0.433 |
| VO₂peak, L/min | Cobb angle (original) | 1.0 | 0.9 | 0.359 |
| VO₂peak, L/min | Thoracic height + Cobb + age + sex | 1.0 | 0.9 | 0.338 |
| VO₂peak, mL/min/kg | Thoracic height + age + sex (primary) | 21.1 | 23.0 | 0.316 |
| VO₂peak, mL/min/kg | Cobb angle (original) | 20.9 | 23.4 | 0.202 |
| VO₂peak, mL/min/kg | Thoracic height + Cobb + age + sex | 21.3 | 22.8 | 0.426 |
| AT, mL/min/kg | Thoracic height + age + sex (primary) | 14.2 | 14.2 | 0.985 |
| AT, mL/min/kg | Cobb angle (original) | 13.7 | 15.0 | 0.297 |
| AT, mL/min/kg | Thoracic height + Cobb + age + sex | 14.2 | 14.2 | 0.980 |
| O₂ pulse %pred | Thoracic height + age + sex (primary) | 71.9 | 74.3 | 0.782 |
| O₂ pulse %pred | Cobb angle (original) | 72.2 | 73.8 | 0.846 |
| O₂ pulse %pred | Thoracic height + Cobb + age + sex | 72.3 | 73.7 | 0.869 |

## Supplementary Table S5. Spirometric percent-predicted values with alternative reference equations (20 controls vs 33 patients)

| **Reference equation** | **Index** | **Control** | **Scoliosis** | **q (FDR)** | **Hedges g** |
| --- | --- | --- | --- | --- | --- |
| GLI Global 2022 (primary) | FVC %pred | 92.82 ± 10.02 | 71.97 ± 28.14 | <0.001 | 0.89 |
| GLI Global 2022 (primary) | FEV₁ %pred | 88.37 ± 10.72 | 70.33 ± 28.26 | 0.002 | 0.76 |
| Jian 2017 (Chinese) | FVC %pred | 91.18 ± 9.57 | 70.02 ± 27.80 | <0.001 | 0.91 |
| Jian 2017 (Chinese) | FEV₁ %pred | 85.38 ± 10.33 | 67.25 ± 27.27 | 0.001 | 0.79 |
| Device default (Zapletal < 18 y; ECCS ≥ 18 y) | FVC %pred | 90.22 ± 11.47 | 70.15 ± 27.03 | 0.002 | 0.88 |
| Device default (Zapletal < 18 y; ECCS ≥ 18 y) | FEV₁ %pred | 88.71 ± 13.00 | 71.70 ± 28.41 | 0.019 | 0.70 |
| Device default (Zapletal < 18 y; ECCS ≥ 18 y) | VC max %pred | 94.95 ± 13.20 | 77.03 ± 27.77 | 0.004 | 0.75 |

Thoracic-height discrimination of FVC < 80% predicted: AUC 0.90 (cutoff 19.6 cm) with GLI Global, 0.88 (19.6 cm) with Jian 2017, and 0.87 (19.6 cm) with the device equations.
